# Expanding the Therapeutic Profile of Topical Cannabidiol in Temporomandibular Disorders: Effects on Sleep Quality and Migraine Disability in Patients with Bruxism-Associated Muscle Pain

**DOI:** 10.3390/ph18071064

**Published:** 2025-07-19

**Authors:** Karolina Walczyńska-Dragon, Jakub Fiegler-Rudol, Stefan Baron, Aleksandra Nitecka-Buchta

**Affiliations:** 1 Department of Temporomandibular Disorders, Medical University of Silesia in Katowice, 41-800 Zabrze, Poland; sbaron@sum.edu.pl (S.B.); aleksandra.nitecka@sum.edu.pl (A.N.-B.); 2 Scientific Society, Department of Temporomandibular Disorders, Medical University of Silesia in Katowice, 41-800 Zabrze, Poland; s88998@365.sum.edu.pl

**Keywords:** topical cannabidiol, temporomandibular disorders, bruxism, migraine disability, sleep disturbances, surface electromyography, PSQI, MIDAS, muscle pain relief

## Abstract

**Background**: Cannabidiol (CBD) has demonstrated potential as a therapeutic agent for muscle tension, pain, and sleep bruxism, yet its broader impact on comorbid conditions such as sleep disturbance and migraine disability remains underexplored. This study aimed to assess the effects of topical CBD on sleep quality and migraine-related disability in patients with bruxism-associated muscular pain. **Methods**: In a randomized, double-blind clinical trial, 60 participants with bruxism were allocated equally into three groups: control (placebo gel), 5% CBD gel, and 10% CBD gel. Participants applied the gel intraorally to the masseter muscles nightly for 30 days. Sleep quality and migraine-related disability were assessed using the Pittsburgh Sleep Quality Index (PSQI) and the Migraine Disability Assessment Scale (MIDAS), respectively. Surface electromyography (sEMG) and the Bruxoff^®^ device were used for objective evaluation of muscle tension and bruxism intensity. **Results**: Both CBD treatment groups demonstrated statistically significant improvements in PSQI and MIDAS scores compared to the control group (*p* < 0.001). No significant differences were observed between the 5% and 10% CBD groups, suggesting comparable efficacy. The sEMG findings corroborated a reduction in muscle tension. Improvements in sleep and migraine outcomes were positively correlated with reductions in muscle activity and pain. **Conclusions**: Topical CBD gel significantly improved sleep quality and reduced migraine-related disability in patients with bruxism-associated muscular pain, supporting its role as a multifaceted therapeutic option in the management of TMD and related comorbidities. Further research is needed to confirm long-term benefits and determine optimal dosing strategies.

## 1. Introduction

### 1.1. Rationale

Temporomandibular disorders (TMDs) are a group of musculoskeletal and neuromuscular conditions affecting the temporomandibular joint (TMJ), masticatory muscles, and associated structures [1,2,3,4,5,6,7,8,9,10]. These disorders are characterized by chronic orofacial pain, joint sounds, and limited jaw mobility and often co-occur with parafunctional habits such as bruxism, repetitive clenching or grinding of the teeth, particularly during sleep [10,11,12]. Bruxism has been linked not only to mechanical wear and muscle fatigue but also to broader physiological dysregulations, including sleep fragmentation, heightened sympathetic activity, and chronic pain syndromes [11,12,13,14]. Importantly, individuals with TMD and bruxism frequently report comorbid conditions such as insomnia and migraine headaches, suggesting overlapping pathophysiological mechanisms involving central sensitization, muscle hyperactivity, and stress-related neurochemical pathways [13,15,16,17]. Cannabidiol (CBD), a non-psychoactive phytocannabinoid derived from Cannabis sativa, has emerged as a promising compound for the management of chronic pain and associated symptoms. Unlike Δ9-tetrahydrocannabinol (THC), CBD does not produce intoxicating effects, and its safety profile has led to increased interest in both topical and systemic formulations [18,19,20,21,22,23,24,25]. Mechanistically, CBD interacts with multiple receptor systems, including cannabinoid receptors CB1 and CB2, serotonin receptors (e.g., 5-HT1A), and transient receptor potential vanilloid channels (e.g., TRPV1) [26,27,28,29,30]. These interactions modulate nociceptive signaling, neuroinflammation, and muscle tone regulation, making CBD relevant for conditions characterized by pain, sleep dysfunction, and neuromuscular hyperactivity [31,32,33,34,35]. A growing body of evidence supports the analgesic, anti-inflammatory, anxiolytic, and sleep-regulating effects of CBD. In animal models, CBD has demonstrated efficacy in reducing nociceptive thresholds, thermal hyperalgesia, and mechanical allodynia, with benefits observed in both inflammatory and neuropathic pain paradigms [36,37,38]. Clinical studies suggest that oral or sublingual CBD (doses ranging from 25 to 300 mg/day) may reduce migraine frequency, improve sleep onset latency, and enhance overall sleep efficiency [26,30,33]. In topical formulations, CBD offers the advantage of localized delivery with minimal systemic absorption, which is particularly desirable in managing focal muscular pain, such as that found in TMD and bruxism. Recent findings from our group [5] have shown that topical application of a 10% CBD gel to the masseter muscles in patients with sleep bruxism leads to significant reductions in muscle activity and subjective pain intensity. Using surface electromyography (sEMG) and the visual analog scale (VAS), we observed that nightly intraoral application of the gel decreased resting muscle tension and improved patient-reported outcomes related to orofacial discomfort. However, while those results established the local muscle-relaxant and analgesic properties of topical CBD, the broader systemic impact, particularly on comorbid sleep disturbance and migraine disability, remains inadequately explored. Given the established comorbidity between TMD, bruxism, insomnia, and migraine and the growing evidence for CBD’s multimodal actions, this study aimed to expand the therapeutic profile of topical CBD by evaluating its effects not only on muscle pain but also on sleep quality and headache-related functional impairment. A better understanding of these interconnected pathways could offer a more holistic approach to the management of bruxism-associated TMD and its frequently overlapping symptoms.

### 1.2. Objectives

The primary objective of this study was to assess the effects of topical CBD gel on sleep quality and migraine-related disability in patients with bruxism-associated muscular pain. Specifically, this research sought to determine whether reductions in muscle tension and pain following CBD treatment correlate with improved sleep quality as measured by the Pittsburgh Sleep Quality Index (PSQI); evaluate whether decreased bruxism intensity is associated with improved Migraine Disability Assessment Scale (MIDAS) scores; and examine the relationships between bruxism, sleep disturbance, and headache burden in the context of CBD therapy. Through a combination of subjective psychometric tools (PSQI, MIDAS) and objective assessments (sEMG; Neurobit Systems, Gdynia, Poland, Bruxoff® recordings; Spes Medica, Genova, Italy), we aimed to characterize the broader therapeutic profile of topical CBD and its potential to address multiple facets of dysfunction in patients with temporomandibular disorders.

## 2. Results

### 2.1. Baseline Demographic and Clinical Characteristics of Participants

**Figure 1:**
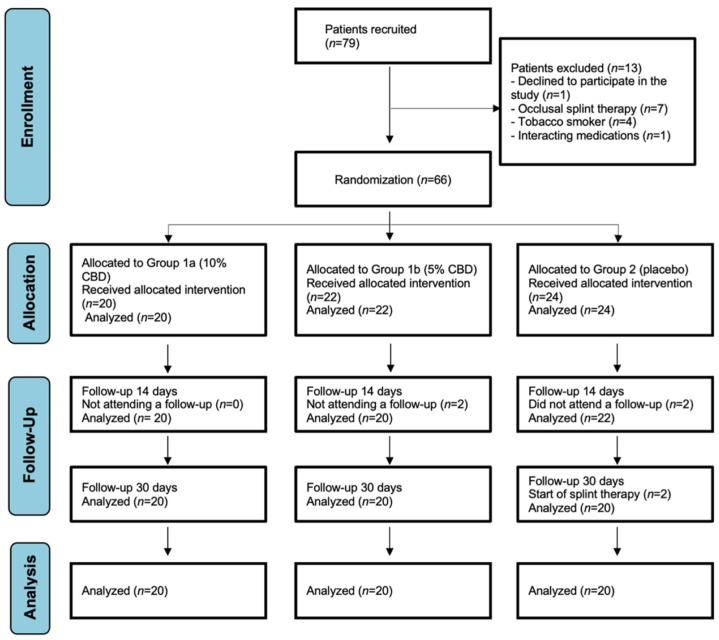
CONSORT flow diagram illustrating the progression of the study participants.

Table 1 presents the baseline characteristics of the study participants divided into three groups: Group 1a (10% CBD), Group 1b (5% CBD), and Group 2 (Placebo), each comprising 20 individuals. The average age across groups was similar, ranging from 23.4 to 24.2 years, with a total mean age of 23.87 years. The sex distribution was relatively balanced, with females representing 60% of the total cohort. Pain intensity, measured using the VAS, was consistent across groups at baseline (day 0), with a mean score of 6.0. By day 14, VAS scores decreased in all groups, with greater reductions in the CBD groups (Group 1a: 4.0; Group 1b: 4.5) compared to the placebo (5.0). By day 30, the reduction in pain was most pronounced in Group 1a (2.0), followed by Group 1b (3.5), while the placebo group showed minimal improvement (5.5), suggesting a potential dose-dependent effect of CBD on pain reduction. Figure 1 shows the CONSORT flow chart of the study participants. A post hoc power analysis confirmed that the actual achieved sample size (*n* = 20 per group) yielded adequate power (≥0.80) to detect the significant improvements observed in sleep quality (PSQI) and migraine disability (MIDAS).

### 2.2. Sleep Quality (PSQI)

**Figure 2:**
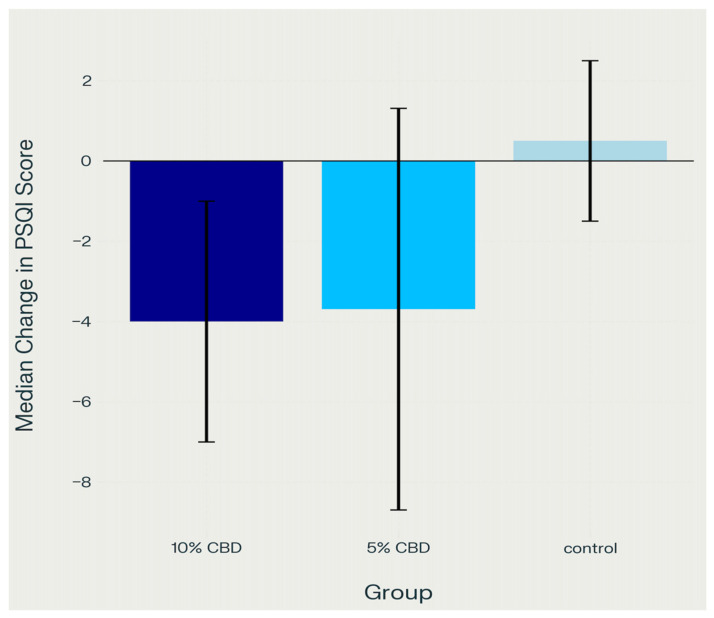
Bar chart showing that both the 5% and 10% CBD groups experienced a median decrease in PSQI scores, indicating improved sleep quality, while the control group showed a slight increase, with the 10% CBD group demonstrating the greatest median improvement.

The Kruskal–Wallis test revealed statistically significant differences among groups for both the PSQI (test statistic = 17.502, *p* < 0.001) and MIDAS (test statistic = 18.233, *p* < 0.001) measures. To further analyze differences between groups, post hoc comparisons were conducted using the Mann–Whitney U test. This indicates that at least one of the compared groups (control, 5% CBD, 10% CBD) exhibited a statistically significant difference in the assessed patient functions (Figure 2). 

Table 2 presents the mean PSQI scores (IQR) at baseline, day 14, and day 30 for each group. Both CBD groups showed a reduction in PSQI over time, indicating improved sleep quality. Notably, the 10% CBD gel group demonstrated the greatest numerical decrease from baseline to day 30 (from a median of 12.0 [IQR 9.0–14.0] to 7.0 [IQR 6.0–9.0]). The 5% CBD group also showed improvement, though slightly less pronounced. In contrast, the control group exhibited minimal change in PSQI scores over the 30-day period.

### 2.3. Migraine Disability (MIDAS)

**Figure 3:**
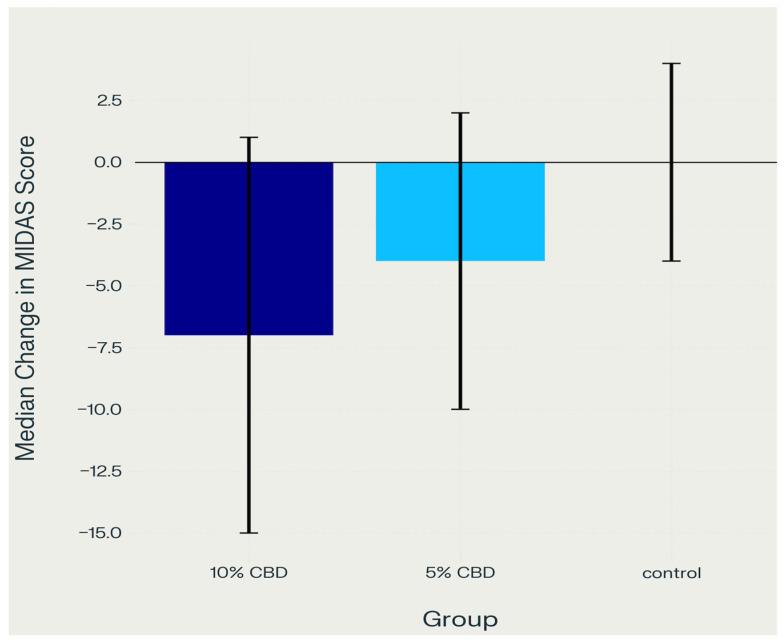
Both the 5% and 10% CBD groups experienced a median reduction in MIDAS scores, indicating decreased migraine-related disability, with the 10% CBD group showing the greatest improvement, while the control group showed minimal change.

Further analyses revealed statistically significant differences between treatment groups. The comparison between the control group and the 5% CBD group showed a significant improvement in daily functioning related to migraines with the use of 5% CBD gel (*p* = 0.01). An even stronger effect was observed in the 10% CBD group compared to the control group (*p* < 0.001), indicating greater reduction in migraine-related functional limitations. However, there was no statistically significant difference between the 5% and 10% CBD groups (*p* = 0.185), suggesting that increasing the CBD concentration from 5% to 10% did not yield additional significant improvements in patient functioning. Post hoc test results assessing the effect of CBD gel on MIDAS scores demonstrated significant differences between certain groups. The comparison between the control group and the 5% CBD group showed a mean rank difference of −12.829 with a standard error of 4.972, yielding a standardized test statistic of −2.58 and a statistically significant *p*-value of 0.01. A greater effect was observed between the control group and the 10% CBD group, with a mean rank difference of −19.842, a standard error of 4.767, a test statistic of −4.162, and a highly significant *p*-value of less than 0.001 (Table 3). However, the comparison between the 5% CBD and 10% CBD groups resulted in a mean rank difference of −7.013, a standard error of 5.289, and a test statistic of −1.326, with a non-significant *p*-value of 0.185, indicating no meaningful difference between these two concentrations (Figure 3).

MIDAS scores were recorded at baseline and day 30 (Table 4). The 10% CBD group showed the most substantial decline in MIDAS scores, decreasing from a median of 17.0 [IQR 12.0–22.0] at baseline to 8.0 [IQR 6.0–10.0] at day 30, suggesting a marked reduction in migraine-related disability over the 30-day treatment period. The 5% CBD group also improved, with scores decreasing from 15.0 [IQR 10.0–19.0] to 10.0 [IQR 7.0–13.0], albeit to a lesser extent. In contrast, the control group’s MIDAS scores changed only minimally, from 12.0 [IQR 8.0–15.0] to 12.0 [IQR 8.0–15.0].

### 2.4. Summary of Key Findings

Across all measures, both CBD groups demonstrated clear improvements in sleep quality and migraine-related disability compared to controls. In the 10% CBD group, PSQI scores declined from a median of 12.0 [IQR 9.0–14.0] at baseline to 10.0 [IQR 8.0–12.0] by day 14 and 7.0 [IQR 6.0–9.0] by day 30, alongside a notable reduction in MIDAS scores from 17.0 [IQR 12.0–22.0] to 8.0 [IQR 6.0–10.0]. The 5% CBD group also improved, with PSQI scores decreasing from 12.0 [IQR 9.0–14.0] to 11.0 [IQR 9.0–13.0] at day 14 and 8.0 [IQR 6.0–10.0] at day 30, while MIDAS scores dropped from 15.0 [IQR 10.0–19.0] to 10.0 [IQR 7.0–13.0]. In contrast, the control group exhibited only minor or no clinically meaningful changes in PSQI (remaining at 9.0 [IQR 6.0–11.0]) or MIDAS scores (12.0 [IQR 8.0–15.0] at both time points). These findings suggest that topical CBD gel, particularly at higher concentrations, may confer additional therapeutic benefits by improving sleep quality and reducing migraine burden in individuals with bruxism-associated muscular pain. Supporting this, statistical analysis using the Kruskal–Wallis test revealed significant between-group differences for both the PSQI (*p* < 0.001) and MIDAS (*p* < 0.001), indicating that CBD gel had a statistically significant impact on sleep quality and migraine-related disability.

#### 2.4.1. Sleep Quality (PSQI)

The analysis revealed significantly lower PSQI values in both CBD groups compared to the control group:

- Control group vs. 5% CBD: *p* = 0.002
- Control group vs. 10% CBD: *p* < 0.001.

No significant difference was observed between the 5% and 10% CBD groups (*p* = 0.602), suggesting that a higher CBD concentration does not provide additional benefits in improving sleep quality.

#### 2.4.2. Migraine Disability (MIDAS)

Similar to the PSQI, CBD application was associated with significant improvement in MIDAS scores compared to the control group:

- Control group vs. 5% CBD: *p* = 0.010
- Control group vs. 10% CBD: *p* < 0.001.

No significant difference was observed between the 5% and 10% CBD groups (*p* = 0.185), indicating that both concentrations exhibit a comparable impact on reducing migraine-related disability.

### 2.5. Safety and Adverse Events

No serious adverse events or treatment-related complications were reported during the study period. All participants completed the 30-day intervention without discontinuation or withdrawal due to side effects. Participants were monitored at each scheduled visit (day 0, day 14, and day 30) for local adverse effects, including signs of oral mucosal irritation, burning, swelling, or lesions. Additionally, they were asked to report any systemic symptoms such as dizziness, sedation, gastrointestinal upset, or allergic reactions. No participants in any group reported adverse events related to the study gels. The treating physician also conducted a visual inspection of the application site at each visit and found no signs of mucosal damage or hypersensitivity reactions. These findings suggest that topical CBD gel, at both 5% and 10% concentrations, was well tolerated over the 30-day application period.

### 2.6. VAS and sEMG Results

In addition to improvements in sleep quality and migraine-related disability, objective and subjective assessments from a parallel randomized clinical trial support the myorelaxant and analgesic effects of topical CBD. In that study, patients receiving intraoral 10% CBD gel exhibited a 42.1% reduction in masseter muscle activity, as measured by surface electromyography (sEMG), alongside a 57.4% decrease in pain intensity reported on the visual analog scale (VAS). The 5% CBD formulation also yielded significant, though less pronounced, effects, with a 40.8% reduction in VAS scores and notable declines in sEMG values. In contrast, the placebo group showed no significant change in either parameter. These results substantiate the muscle-relaxing and analgesic mechanisms proposed in our findings and reinforce the role of CBD in reducing muscle hyperactivity and nociceptive input in temporomandibular disorder (TMD) patients with bruxism.

## 3. Discussion

This study provides novel evidence that topical application of cannabidiol (CBD) gel can significantly improve sleep quality and reduce migraine-related disability in individuals with bruxism-associated muscular pain. These findings extend prior research [5,26,33] by demonstrating that CBD’s therapeutic benefits are not limited to localized pain relief but also extend to systemic symptoms commonly comorbid with temporomandibular disorders (TMD), such as insomnia and migraines. This outcome supports the hypothesis that bruxism, sleep disturbances, and headaches share overlapping neurophysiological mechanisms that may be modulated through targeted cannabinoid intervention [15,16,17]. The interplay between TMD, bruxism, and systemic symptoms is complex. Sleep bruxism is associated with increased muscle tone and sympathetic nervous system activity, both of which contribute to pain amplification and sleep fragmentation [10,12,13]. TMD patients frequently report poor sleep quality and recurrent headaches, which exacerbate pain perception and contribute to a cycle of chronic discomfort [14,15,23]. By reducing muscle hyperactivity and tension—as shown by significant reductions in surface electromyography (sEMG) values and pain levels (VAS)—CBD may interrupt this cycle and promote downstream improvements in sleep and headache outcomes. Importantly, the route of administration appears to be a critical factor in achieving these results. The topical intraoral application of CBD in this study allowed for localized targeting of the masseter muscles, which are heavily involved in bruxism and orofacial pain [5,12]. Unlike oral or systemic cannabinoid formulations, which are associated with variable bioavailability and potential central side effects, topical delivery provides a controlled and peripheral action that minimizes systemic exposure while maximizing localized therapeutic impact [31,32,33]. The daily dose of 25–50 mg CBD per application falls within the lower range of clinically used topical doses and showed efficacy in modulating both subjective (PSQI, MIDAS) and objective (sEMG, bruxism index) outcomes. These findings align with a growing body of the literature supporting the analgesic, anti-inflammatory, anxiolytic, and sleep-regulating properties of CBD [1,2,3,4,6,7,8,26,27,28,29,30]. In preclinical studies, CBD has been shown to reduce hyperalgesia, allodynia, and muscle sensitivity via activation of TRPV1 channels and CB1/CB2 receptor modulation [3,6,38]. Clinical data suggest that cannabinoids may reduce migraine frequency, improve sleep latency, and reduce anxiety-related muscle tension [29,30,33]. The results from our study further suggest that these effects can be achieved through topical delivery in a localized region, with broader systemic benefits mediated indirectly through improved neuromuscular function and pain relief. One particularly important observation is that no significant difference was found between the 5% and 10% CBD gel groups in terms of sleep or migraine improvement. Despite these encouraging findings, the study has limitations. First, the treatment duration was limited to 30 days, and it remains unclear whether the benefits are sustained over time or diminish post-treatment. Second, sleep outcomes were assessed using the PSQI, a validated but subjective tool. Future studies should consider integrating objective sleep assessments such as polysomnography or actigraphy. Third, although the study controlled for many confounders, the use of self-reported pain and headache measures remains vulnerable to response bias. While no statistically significant differences were observed between the 5% and 10% CBD groups, the limited number of dosage levels prevents a definitive conclusion about the dose–response relationship. Additionally, while the topical application was well tolerated, we did not conduct pharmacokinetic evaluations, so systemic absorption, albeit likely minimal, was not quantified. Another notable consideration is the biopsychosocial complexity of TMD and bruxism. While our initial sample size calculation underestimated the required number of participants per group, post hoc analysis indicated sufficient statistical power to support our findings. Nonetheless, future studies should consider recruiting larger sample sizes to confirm and potentially extend these observations. This study did not apply corrections for multiple statistical comparisons, which increases the risk of type I error. The findings, particularly from the post hoc analyses, should therefore be interpreted with caution. While CBD may reduce physiological symptoms, psychological and behavioral components, such as stress, anxiety, and poor sleep hygiene, also contribute significantly to symptom severity and treatment outcomes [17,30]. A multimodal management plan that incorporates behavioral therapy, sleep hygiene education, and physical rehabilitation may further amplify the therapeutic gains achieved with CBD. In conclusion, our findings support the use of topical CBD as a promising adjunctive treatment for individuals with TMD and bruxism-associated muscle pain. The improvements observed in sleep quality and migraine disability reflect a broader systemic effect likely mediated through the reduction in local muscle tension and pain. Given its non-invasive, localized, and well-tolerated profile, topical CBD may be especially useful in patients with multifactorial symptom presentations, particularly where pain, sleep disturbance, and migraine overlap. Future studies should explore optimal dosing strategies, long-term effects, and mechanistic pathways, ideally incorporating objective neurophysiological and sleep measures to validate and expand upon these preliminary findings. To aid interpretation of the clinical relevance of the observed improvements, we considered minimal clinically important differences (MCID) reported in the literature. For the PSQI, a change of at least three points is generally regarded as clinically meaningful in populations with sleep disturbance. In our study, the 10% CBD group improved by a median of five points over 30 days, exceeding this threshold. Similarly, for the MIDAS scale, although MCID thresholds are less clearly established, a reduction of at least five points has been suggested as indicative of a meaningful improvement in functional impairment. Both CBD groups met or exceeded this benchmark, suggesting that the observed effects are not only statistically significant but also clinically relevant.

## 4. Materials and Methods

### 4.1. Study Design

This study was conducted as a parallel-group, three-arm, randomized, double-blind clinical trial with an equal 1:1:1 allocation ratio. Participants were recruited from patients referred to the Department of Temporomandibular Disorders at the Medical University of Silesia, Poland.

### 4.2. Participant Recruitment and Sample Size

This study was designed as a parallel-group, three-arm, randomized, double-blind clinical trial with a 1:1:1 allocation ratio. Participants were recruited from patients referred to the Department of Temporomandibular Disorders at the Medical University of Silesia, Poland. A formal a priori power analysis was conducted to determine the required sample size based on expected changes in two primary outcomes: the PSQI and the MIDAS, assessed before and after the intervention. A formal a priori power analysis was initially performed to estimate the required sample size based on an anticipated large effect size (Cohen’s d = 0.8), informed by pilot studies and previously published studies demonstrating substantial effects of topical cannabidiol (CBD) on muscular tension, pain, and sleep quality [5,26,33]. However, recalculation using the G*Power software (version 3.1.9.7), with the same parameters (Cohen’s d = 0.8; α = 0.05; power = 0.80; two-tailed), indicated that approximately 26 participants per group would have been required rather than the 20 participants initially stated. Due to practical constraints and dropouts, the final sample size was 20 participants per group, slightly below the recalculated recommendation. Consequently, a post hoc power analysis was conducted, confirming that the actual sample size still provided adequate power (≥0.80) to detect the observed significant differences in primary outcomes. Table 5 shows the inclusion and exclusion criteria used in this study.

### 4.3. Study Groups

Participants were randomly allocated into three groups (*n* = 20 each):

- Group 1 (control group) received a placebo gel without cannabidiol.
- Group 2 (CBD 5% group) received a 5% CBD hydrogel.
- Group 3 (CBD 10% group) received a 10% CBD hydrogel.

All gels were applied intraorally to the masseter muscle region once nightly for 30 days.

### 4.4. Ethical Considerations and Registration

The study received approval from the Bioethical Committee of the Medical University of Silesia (approval number PCN/0022/KB1/66/II/20/21, granted on 20 April 2021) and was prospectively registered on ClinicalTrials.gov (NCT05562635) (accessed on 31 August 2022). The research was conducted in accordance with the principles outlined in the Declaration of Helsinki and the International Conference on Harmonization guidelines for Good Clinical Practice. Prior to participation, all patients received both oral and written information regarding the study and provided informed consent. A total of 60 participants (20 per group) completed the study.

### 4.5. Study Protocol

During the scheduled visits, participants first attended a screening visit where they underwent an initial qualification assessment and allergy screening for the CBD formulation, and their sleep bruxism intensity was measured using the bruxism index (BRK), which quantified the number of bruxism episodes per hour; they were also provided with a Bruxoff device for home monitoring. At the baseline visit (day 0), participants underwent their first surface electromyography assessment (sEMG I), rated their pain levels using the visual analogue scale (VAS I), and were randomized into respective study groups, with additional assessments conducted using the Pittsburgh Sleep Quality Index (PSQI) and the Migraine Disability Assessment Scale (MIDAS). The first follow-up visit, conducted on day 14 after 14 days of CBD gel application, included a repeat PSQI sleep assessment, a second sEMG evaluation (sEMG II), and pain evaluation using VAS II. The second follow-up visit on day 30 involved a final sEMG assessment (sEMG III), another pain evaluation (VAS III), a repeat Bruxoff examination, and final assessments using the PSQI and MIDAS scales. Patients completed the PSQI three times: at the baseline visit, after 14 days, and after 30 days. The MIDAS was evaluated twice, at the baseline visit and after 30 days. All questionnaires were administered in a controlled clinical setting, in the same location and in the presence of a physician, ensuring standardized conditions. To minimize bias, participants remained blinded to their group allocation (1a, 1b, or 2).

#### Randomization and Blinding

Participants were randomly assigned to one of three study groups (placebo, 5% CBD, or 10% CBD gel) using a computer-generated randomization sequence prepared by an independent researcher not involved in recruitment or evaluation. Group allocation followed a simple randomization protocol with a 1:1:1 ratio. Each participant was assigned a unique identification number corresponding to pre-coded sealed opaque envelopes, which were opened by the clinical staff only after completion of the baseline evaluation. Blinding procedures were applied to both participants and evaluating physicians. The investigator responsible for administering the PSQI, MIDAS, VAS, and sEMG assessments was blinded to group allocation, as was the statistician conducting the data analysis. Gels were dispensed in identical, unmarked Uno Dose containers, labeled only with the participant’s ID. The containers were indistinguishable in shape and external appearance. To maintain blinding integrity, the base hydrogel formulation (placebo) and the CBD-containing gels (5% and 10%) were manufactured using the same hydroxyethyl cellulose matrix with paraffin oil as the dispersing agent. While the placebo gel was transparent, the 5% and 10% CBD gels had a slightly white or opaque appearance due to the inclusion of crystalline CBD isolate. All gels had a similar viscosity and texture, and no scenting agents were used. Although minor visual differences existed, neither participants nor clinicians were informed of these characteristics, and no participants reported awareness of group identity during the study.

### 4.6. Experimental Gel Preparation and Application

The experimental gel for Groups 1a and 1b was prepared by combining 100% CBD isolate in powder form with a hydrogel base composed of hydroxyethyl cellulose (Celugel, Actifarm, Permit No 30050). The gel was formulated in two concentrations, 5% and 10% CBD, with paraffin oil facilitating the grinding of CBD powder. The mixture was processed in an Eprus^®^ U500 automatic recipe mixer and dispensed into Uno Dose containers (Eprus PN-EN ISO 15378:2018, Newark, NJ, USA) [39], ensuring accurate daily dosing. Patients applied the gel intraorally to the masseter muscles before bedtime, avoiding food or drink intake afterward. The control group received a polymer gel without CBD. The 5% and 10% CBD gels were selected based on previous preclinical and clinical studies demonstrating safety and efficacy within this concentration range for topical application [5,31,32,33]. Each unit-dose container (Uno Dose, Eprus^®^ PN-EN ISO 15378:2018, Newark, NJ, USA) [39] delivered a fixed volume of 0.5 mL of gel per application. This corresponds to a single dose of 25 mg CBD for the 5% group and 50 mg CBD for the 10% group. Patients were instructed to apply the entire contents of one container directly to the inner cheek and over the masseter muscle region bilaterally using clean hands, every evening before bedtime, and to refrain from eating or drinking afterward.

### 4.7. Assessment Methods

#### 4.7.1. Bruxism Assessment

Bruxism assessment was conducted using the Bruxoff^®^ device (Spes Medica, Genova, Italy), which recorded surface electromyography (sEMG) and electrocardiography (ECG) signals. Patients self-applied disposable surface electrodes to the masseter muscles and thorax under clinician guidance. The device captured bruxism indices, mean heart rate, and muscle contractions (tonic, phasic, and mixed). Automated scoring was performed using the Bruxmeter software v.2.0.2.7 (OT Biolettonica, Torino, Italy).

#### 4.7.2. Muscle Tension (sEMG)

Muscle tension was measured with the Neurobit Optima 4.0 system (Neurobit Systems, Gdynia, Poland), recording resting and maximal voluntary contraction (MVIC) sEMG values. Electrodes were placed bilaterally over the masseter muscles following SENIAM guidelines. Patients completed three MVIC tests with a one-minute rest interval, and the results were normalized and analyzed. Measurements were performed at baseline (day 0), after 14 days, and at 30 days.

#### 4.7.3. Pain Levels (VAS)

Pain levels were assessed at each visit using the visual analog scale (VAS), ranging from 0 (no pain) to 10 (worst pain).

#### 4.7.4. Sleep Quality (PSQI)

The Pittsburgh Sleep Quality Index assessment was conducted three times: at baseline, after 14 days, and after 30 days of CBD or placebo gel application in all three groups. The PSQI categorizes sleep disturbances into four levels, ranging from good sleep quality to severe disturbances requiring medical attention. The following classification provides a detailed breakdown of the PSQI scoring system:

- 0–5 points: Good sleep quality (no significant problems)
- 6–10 points: Mild sleep disturbances (may stem from lifestyle, stress, or poor sleep habits)
- 11–15 points: Moderate sleep disturbances (suggesting possible chronic issues, e.g., insomnia or excessive daytime sleepiness)
- 16–21 points: Severe sleep disturbances (indicating serious problems requiring medical intervention, e.g., sleep apnea, chronic insomnia, circadian rhythm disorders).

#### 4.7.5. Migraine Disability (MIDAS)

The Migraine Disability Assessment Scale evaluation was performed twice, at the baseline visit and after 30 days of CBD or placebo gel use. The MIDAS scale categorizes headache disability into four grades, ranging from minimal to severe, based on the extent to which migraines interfere with daily activities. The following classification outlines the scoring system:

- 0–5 points (Grade I)—No or minimal disability;
  ○ Migraine has negligible impact on daily life;
- 6–10 points (Grade II)—Mild disability;
  ○ Migraine occasionally interferes with daily functioning;
- 11–20 points (Grade III)—Moderate disability;
  ○ Migraine significantly affects work and daily life;
- ≥21 points (Grade IV)—Severe disability;
  ○ Severe restriction of daily activities; requires advanced treatment.

### 4.8. Statistical Analysis

Statistical analyses were conducted to evaluate the impact of cannabidiol (CBD) gel application on sleep quality and migraine-related disability in patients with bruxism-associated muscular pain. Prior to group comparisons, data normality was assessed using the Shapiro–Wilk test, which indicated deviations from normal distribution in at least one subgroup. Accordingly, non-parametric tests were applied. The Kruskal–Wallis test was used to identify global differences across the three study groups (control, 5% CBD, and 10% CBD) for each outcome variable, including the Pittsburgh Sleep Quality Index and the Migraine Disability Assessment Scale. Where significant differences were found, post hoc pairwise comparisons were performed using the Mann–Whitney U test to determine specific group differences. Statistical significance was set at *p* < 0.05. Given the multiple pairwise comparisons performed, we acknowledge that no formal adjustment (e.g., Bonferroni’s correction) was applied to control for type I error, which represents a limitation of the statistical approach.

### 4.9. Safety Monitoring

Participants were evaluated for adverse effects during each scheduled visit. Safety assessments included patient self-reporting of any local (e.g., mucosal irritation, burning, swelling) or systemic symptoms (e.g., dizziness, drowsiness, gastrointestinal discomfort), as well as clinical examination of the intraoral application area. Any adverse events were to be documented and reported to the ethics committee. No participants used prohibited medications during the trial period, and no rescue analgesics were permitted.

## 5. Conclusions

This study demonstrates that topical application of cannabidiol (CBD) gel, at both 5% and 10% concentrations, significantly improves sleep quality and reduces migraine-related disability in patients with bruxism-associated muscular pain. These effects were observed alongside reductions in muscle tension and pain, suggesting a broader therapeutic impact of CBD beyond localized symptom relief. Notably, no substantial differences were found between the two concentrations, indicating that lower doses may achieve comparable clinical outcomes. The findings support the use of topical CBD as a well-tolerated, non-invasive adjunct in the multimodal management of temporomandibular disorders (TMD), especially in patients experiencing comorbid sleep and headache disturbances. Future research should explore long-term efficacy, optimal dosing, and underlying mechanisms through objective neurophysiological and sleep assessments.

## Figures and Tables

**Table 1:** Baseline demographic and clinical characteristics of participants.

Characteristic	Group 1a (10% CBD) (*n* = 20)	Group 1b (5% CBD) (*n* = 20)	Group 2 (Placebo) (*n* = 20)	Total (*n* = 60)
Age, years	24.2	24.0	23.4	23.87
Sex (%)				
Female	13 (65%)	11 (55%)	12 (60%)	36 (60%)
Male	7 (35%)	9 (45%)	8 (40%)	24 (40%)
VAS score (mean, SD)				
Day 0	6.0 (2.0)	6.0 (2.0)	6.0 (3.5)	6.0 (3.0)
Day 14	4.0 (2.0)	4.5 (3.0)	5.0 (2.5)	4.0 (2.0)
Day 30	2.0 (3.0)	3.5 (2.0)	5.5 (3.0)	4.0 (3.0)

**Table 2:** Pittsburgh Sleep Quality Index (PSQI) scores by group and time points.

Group	Baseline (Median [IQR])	Day 14 (Median [IQR])	Day 30 (Median [IQR])
10% CBD	12.0 [9.0–14.0]	10.0 [8.0–12.0]	7.0 [6.0–9.0]
5% CBD	12.0 [9.0–14.0]	11.0 [9.0–13.0]	8.0 [6.0–10.0]
Control	9.0 [6.0–11.0]	9.0 [6.0–12.0]	9.0 [6.0–11.0]

**Table 3:** Post hoc test statistics—effect of CBD gel on MIDAS scale.

Comparison	Mean Rank Difference	Standard Error	Standardized Test Statistic	*p*-Value
Control Group vs. 5% CBD	−12.829	4.972	−2.58	0.01
Control Group vs. 10% CBD	−19.842	4.767	−4.162	<0.001
5% CBD vs. 10% CBD	−7.013	5.289	−1.326	0.185

**Table 4:** Migraine Disability Assessment Scale (MIDAS) scores at baseline and day 30.

Group	Baseline (Median [IQR])	Day 30 (Median [IQR])
10% CBD	17.0 [12.0–22.0]	8.0 [6.0–10.0]
5% CBD	15.0 [10.0–19.0]	10.0 [7.0–13.0]
Control	12.0 [8.0–15.0]	12.0 [8.0–15.0]

**Table 5:** Inclusion and exclusion criteria for the study.

Inclusion Criteria	Exclusion Criteria
Individuals who provided informed consent to participate in the study. Age between 18 and 60 years. Overall good systemic health. Diagnosed with temporomandibular disorders (TMDs) based on the Polish adaptation of the Research Diagnostic Criteria for Temporomandibular Disorders (DC/TMD), specifically Group I, subcategories I.1a, I.1b, and I.1c. Full dentition present, excluding third molars.	Known allergy or hypersensitivity to cannabis-based products, placebo substances, or any study-related compounds. Presence of lesions or open wounds in the oral mucosa. History of cannabis dependence or substance misuse. Active tobacco use. Current use of painkillers or medications that influence muscle activity. Use of intraoral devices such as occlusal appliances. Presence of fixed or removable dental prostheses. Diagnosed with systemic or autoimmune conditions causing generalized muscle tension. Ongoing orthodontic treatment. Under care of a neurologist for neurological disorders. Diagnosed psychiatric conditions. History of radiotherapy or active malignancy. Odontogenic (tooth-related) pain. Pregnant or lactating individuals. Use of medications known to interact with cannabidiol (CBD). Current use of cannabis or cannabis-derived products.

## Data Availability

The original contributions presented in the study are included in the article; further inquiries can be directed to the corresponding author.
